# Vancouver type B2 periprosthetic femoral fractures: clinical and radiological outcomes from a tertiary care center

**DOI:** 10.1007/s00402-023-04955-2

**Published:** 2023-07-01

**Authors:** Stefano Tornago, Luca Cavagnaro, Lorenzo Mosconi, Francesco Chiarlone, Andrea Zanirato, Nicolò Patroniti, Matteo Formica

**Affiliations:** 1 Joint Replacement Unit, Ortopedia e Traumatologia 2, Ospedale Santa Corona, Viale 25 Aprile, 38, 17027 Pietra Ligure, SV Italy; 2 UO 1’ Sezione di Ortopedia e Traumatologia, Istituto Clinico San Rocco Gruppo San Donato, Via dei Sabbioni, 24, BS 25050 Ome, Italy; 3 Policlinico San Martino, Clinica Ortopedica, Largo Rosanna Benzi 10, 16132 Genoa, GE Italy; 4 Anesthesia and Intensive Care Unit, Ospedale Santa Corona, Viale 25 Aprile, 38, 17027 Pietra Ligure, SV Italy; 5 DISC, Dipartimento di Scienze Chirurgiche e Diagnostiche Integrate, Viale Benedetto XV 6, 16132 Genoa, GE Italy

**Keywords:** Periprosthetic fracture, Total hip arthroplasty, Hip revision, Surgical technique, Outcomes

## Abstract

**Introduction:**

The purpose of this study was to report mid-term clinical and radiographic results after hip arthroplasty revision in Vancouver type B2 femoral periprosthetic fractures (PPFx). Specifical focus of the paper is as follows: (1) the description of a standardized and reproducible surgical technique, (2) functional outcomes presentation and (3) type and number of complications and implants’ survival rate analysis.

**Methods:**

We retrospectively reviewed all patients treated for hip revision with non-modular tapered fluted titanium stem in patients with Vancouver type B2 femur PPFx at a single institution. At least 18 months’ follow-up period was required. Harris Hip Scores and SF-12 were obtained, and radiographical follow-up was performed. Complications were reported and analyzed.

**Results:**

The authors included 114 patients (114 hips) with a mean follow-up of 62.8 ± 30.6 months. All patients were treated with Wagner SL revision hip stem (Zimmer-Biomet), metal cerclage wires ± trochanteric plate. The mean HHS and SF-12 score at the last follow-up evaluation were respectively 81.3 ± 9.7 and 32.5 ± 7.6. Seventeen (14.9%) complications occurred. We observed five cases of dislocations, two of periprosthetic joint infections and six cases of new PPFx. The stem-related revision rate for any cause at the final FU was 1.7%, due to PJI. No patients underwent stem revision surgery for aseptic loosening. Fracture healed in all the included patients with a union-rate of 100%. The re-operation rate for any cause was 9.6%, with an implant survival rate for overall failure of 96.5%.

**Conclusion:**

The presented standard and reproducible surgical technique obtains optimal clinical and radiological results with limited complication rate at mid-term follow up. Preoperative planning as well as careful intraoperative surgical technique is of a paramount importance.

## Introduction

Total hip arthroplasty (THA) is a very common procedure routinely performed in the lpast three decades in the aged population, with significant comorbidity and osteopenia. However, now that elderly patients with hip replacements are more and more active with long life expectancy, late periprosthetic fractures (PPFx) around the stem are not unusual. The incidence of PPFx is approximately 4.1%, with higher rates for uncemented stem and revision THA (rTHA) [1, 2] The number is doomed to raise up in the next years, representing a real challenge for the orthopedic surgeons and medical team. In fact, femoral PPFx are burdened by high complication rate, considerable morbidity and dysfunction for patients [3–7]. Moreover, surgical treatment requires both arthroplasty and trauma skills.

A detailed analysis of PPFx, including a classification, is mandatory for defining the best treatment. To our knowledge, Vancouver classification [8] is the most widely used to address the treatment of the different types of PPFx around the femoral stem. It considers the fracture site, the stability of the femoral stem and the quality of surrounding femoral bone stock. The classification has been modified by Masri et al. to include intraoperative in addition to postoperative periprosthetic femur fractures [9] Vancouver classification divides fracture according to the location in type Type A occurring around the trochanteric region, Type B near or just distal to the femoral stem, and Type C well below the femoral stem. Type A is subgrouped in AG (fracture of the greater trochanter) and AL (fracture of the lesser trochanter). Type B fractures are subdivided in B1 (well-fixed stem), B2 (loose stem with good bone stock) and B3 (loose stem with poor bone stock).

Basically, well-fixed stems (Vancouver type B1 and C) need ORIF (open reduction and internal fixation), whereas loose stems (Vancouver type B2 and B3) require femoral implant revision with or without internal fixation [8, 10, 11].The main goals of the surgeon are to achieve fracture reduction with proper alignment and stable fixation, implant stability and patient early mobilization.

The purpose of this study is to report mid-term clinical and radiographic results after hip arthroplasty revision in Vancouver type B2 PPFx. Specifical focus of the paper is: (1) the description of a standardized and reproducible surgical technique (2) functional outcomes presentation, (3) type and number of complications and implants survival rate analysis.

## Material and methods

### Study design

Data of this retrospective single-center study were collected using the prospective institutional arthroplasty registry (Santa Corona Hospital—Pietra Ligure). Written and informed consent was obtained from each individual participant before the revision procedure. The study was approved by the local ethics committee (177/2022) and it was performed in accordance with the ethical standards as laid down in the 1964 Declaration of Helsinki and its later amendments.

The inclusion criteria were hip arthroplasty revision with non-modular tapered fluted titanium stem in patients with Vancouver type B2 femur PPFx, and completion of at least 18 months follow-up (FU) period. Exclusion criteria were Vancouver fractures type A, B1, B3 and C, Vancouver type B2 associated with pelvis fracture. Patients with shorter than 18-months FU were excluded as well. Demographic features of patients and surgical data were collected (Table 1).

**Table 1:** Demographic and surgical data

Sex	
Female	103 (75.2%)
Male	34 (24.8%)
Body mass index (kg/m^2^)	27.6 ± 5.4
Age at time of surgery (year)	79.1 ± 9.2
Side of procedure	
Right	82 (59.8%)
Left	55 (40.2%)
Indication for surgery	
Vancouver type B2 fracture	137 (100%)
ASA score	3.6 ± 0.7
Surgery time (min)	93.8 ± 34.4
Previous stem designed	
Single wedge^a^	46 (33.6%)
Double wedge^a^	68 (49.6%)
Tapered^a^	16 (11.7%)
Anatomic^a^	5 (3.6%)
Cemented	2 (1.5%)
Acetabular revision	15 (10.9%)
Trochanteric plate	5 (3.6%)
*N*° of femoral cerclages/patient	3.6 ± 1.5

^a^Classification of the cementless femoral stem designs (Khanuja et al., 2011)

### Patients

Between January 2011 and June 2020, 137 hip arthroplasty revisions were performed in 137 patients affected by a B2 PPFx of the femur. One hundred and three (75.2%) patients were female and 34 (24.8%) were male. The mean body mass index (BMI) was 27.6 ± 5.4 (range 19.5–34.9) Kg/m^2^ and mean age of the patients at the time of the indexed surgery was 79.1 ± 9.2 (range 41–94) years. The mean ASA scores were 3.2 ± 0.7 (range 2–5). Twenty-three patients were lost at follow up, of these 18 (13.1%) died, with a global drop-out rate of 16.8%. A total of 114 patients were assessed clinically and radiographically for a mean of 62.8 ± 30.6 (range 18–126) months.

In all cases, the indication for the surgery was a PPFx of the femur, type B2 according to the Vancouver classification.

### Surgical technique

**Fig. 1:**
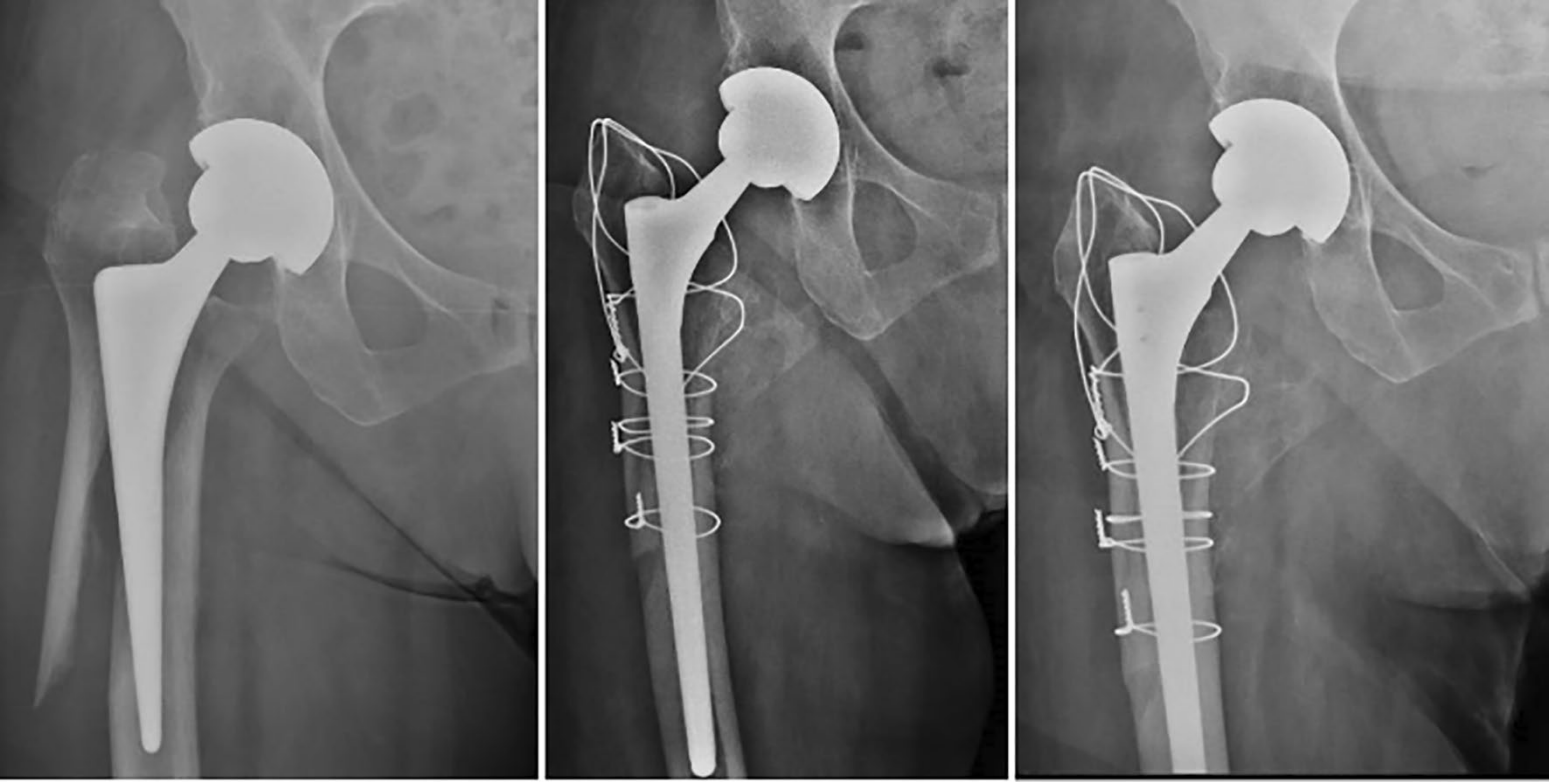
A Vancouver type B2 periprosthetic fracture, treated with stem revision (Wagner SL, Zimmer Biomet), and cerclage wires (2 years follow-up)

Every surgery was performed in a single center, by a senior surgeon (ST, LC) with expertise in hip replacement and revision surgery. In all cases an extended posterolateral approach was performed to allow hip joint exploration and diaphyseal femoral fracture visualization. One or more temporary 2.0 mm cerclage wires were used to reduce and fix the diaphyseal fracture fragments. After the implant dislocation, routinary evaluation of the acetabular component and careful debridement of dead soft tissue and avascularized necrotic bone was performed. Acetabular prosthetic revision was mandatory only in 15 cases. The decision to revise or not the acetabular shell decided was performed case by case by the senior surgeon. Once the femoral head and the loose stem was removed, distal fixation of the uncemented revision stem was obtained. In all cases, the stem bypassed the more distal fracture fragment by al least two cortical femoral diameters to achieve a stable implant fixation [12]. In case of cemented stem loosening, it was removed through suitable osteotomes. Component orientation, leg length discrepancy and implant stability were evaluated intra-operatively after trial component reduction. At the end, the femoral shaft fracture was fully reduced and fixed with definitive metal wires cerclages. In the presence of extension fracture to the great trochanter, a preformed trochanteric plate (in case of comminuted fractures) or dynamic cerclages was performed. Reduction ad fixation of the greater trochanter fracture was always performed with the hip reduced on order to gain the proper soft tissue tension. Figure 1 shows a radiographic analysis of a sample case.

### Post-operative treatment

The rehabilitation program was carried on case by case, considering bone quality, shape of fracture, age and comorbidity of single patient. In general, a partial bearing (50%) with crutches or walker was adopted from the first post-operative day. Antibiotic prophylaxis with Cephalosporin and standard venous thromboembolism prophylaxis with EBPM were administered routinely. In all case 1 drain was used and removed within the second post-operative day.

### Clinical and radiological evaluation

Clinical assessment included physical examination, Harris Hip Score (HHS) and Medical Outcome study short forms 12 (SF-12) were determined routinely at 1, 3 and 12 months after the procedure, and annually thereafter, by senior surgeon. Conventional radiographs (anteroposterior view of the pelvis, anteroposterior and lateral view of the femur) were performed by default at the end of the surgery, at 1, 3, 12 months, and annually thereafter. Radiographic evidence of osseointegration, prosthetic dislocation, stem loosening, osteolysis according to Gruen zones [13], cortical hypertrophy, heterotopic ossification according to Brooker’s classification [14], bone healing or fracture displacement was reviewed by 2 orthopedic surgeons (FC, LM). Doubtful cases were solved by consensus. Complications, revision of fixed components, reoperation and timing from the indexed surgery were recorded. Cerclage removal was not considered as a complication for statistical analysis.

### Statistical analysis

Continuous variables were reported as mean ± standard deviation (SD). Categorical variables were expressed as the number of cases or percentage. Kaplan–Meier survival curves with 95% confidence intervals (CI) were created to analyze femoral implant and fixed components survivorship free of revision for any reason as the endpoints.

## Results

Non-modular tapered fluted titanium stem (Wagner SL revision hip stem; Zimmer-Biomet) was used in all cases. In 80% of the cases, Wagner 225 or 265 was implanted. Main features of the implanted Wagner SL revision stems are summarized in Table 2.

**Table 2:** Length and size of the revision stem (Wagner SL, Zimmer Biomet)

Length stem	*N*° 137 (100%)	Size stem	*N*° 137 (100%)
190	16 (11.7%)	14	34 (24.8%)
225	74 (54.0%)	15	45 (32.8%)
265	38 (27.7%)	16	29 (21.2%)
305	9 (6.6%)	17	16 (11.7%)
		18	10 (7.3%)
		19	3 (2.2%)

Acetabular revision was performed in 15 patients, due to aseptic loosening of the acetabular cup or polyethylene wear without the possibility of replacing the insert (out of business). Liner exchange was performed in 24 (17.6%) patients. In 5 patients with Vancouver type B2 fracture with a comminuted fracture of the greater trochanter, a trochanteric plate was implanted in addition to the replacement of the femoral stem. In 18 patients, the authors performed a trochanteric fracture reduction and fixation with wires. On average, 3.6 ± 1.5 cerclages wires were used for each patient. Although the authors moved to a routine use of ultra-high-molecular-weight polyethylene iso-elastic cables, the case series includes only patients with metallic cerclages. No diaphyseal plates were used to fix the femoral fractures.

In seven (6.1%) patients, during post-operative clinical examination, was observed a leg length discrepancy (LLD), in all cases less than 1 cm.

### Clinical outcomes

The mean HHS and SF-12 score at the last follow-up evaluation were, respectively, 81.3 ± 9.7 and 32.5 ± 7.6. Obviously subjective score was performed only in patient without dementia (subjective scores were not performed in 12 patients). There was only 1 patient during FU period with severe midthigh pain and functional limitation that required re-operation. Seventy-five patients were pain free, 29 patients had occasional pain and the remaining patients had mild pain well managed with painkillers and moderate limited activities of daily living. Globally, 25 patients walked with crouches at final follow-up.

### Radiologic outcome

All radiographic controls showed good AP e LL implant positioning and prosthetic osseointegration without stem loosening, with 16 (14.0%) cases of non-progressive radiolucency in Gruen Zone I–VI–VII. Heterotopic ossifications were observed in 14 (12.3%) patients, without the need to re-operation. Fracture healing was observed in all cases, with 4 (3.5%) displacements of fracture.

### Complications

No intraoperative complications occurred during indexed surgery.

We reported 17 (14.9%) complications that occurred during FU and are summarized in Table 3.

**Table 3:** Post-op complications

Complication	*N*° (%)	Re-operation *N*° (%)
Dislocation	5 (4.4%)	2 (1.7%)
PJI	2 (1.7%)	2 (1.7%)
Recurrence of PPFx	6 (5.3%)	6 (5.3%)
Fracture displacement	4 (3.5%)	1 (5.3%)
Total	17 (14.9%)	11 (9.6%)

Five (4.4%) dislocation were observed. In particular, 3 dislocations occurred during the hospitalization of patients, and in two cases an acetabular revision was performed, using a dual mobility cup; 2 episodes happened at more than 45 days from surgery, were referred as movements exceeding the usual range of motion, and were treated conservatively with good final outcome.

Periprosthetic joint infection (PJI) was seen in 2 (1.7%) patients and were successfully managed with staged revision [15].

Six (5.3%) patients developed new traumatic PPFx, without evidence of radiologic stress shielding or stem loosening. Three of these reported trochanteric fractures, treated with cerclages wires and great trochanteric plate. In the other 3 cases, distal periprosthetic fracture (Vancouver type C) was observed and managed with plate fixation. In all cases, Wagner stems were well fixed and osseointegrated.

Last, we reported 4 displacements of fracture (2 at 30 days and 2 at 90 days after the indexed procedure) that required re-operation in 1 case with additional wires cerclages for severe thigh pain.

**Fig. 2:**
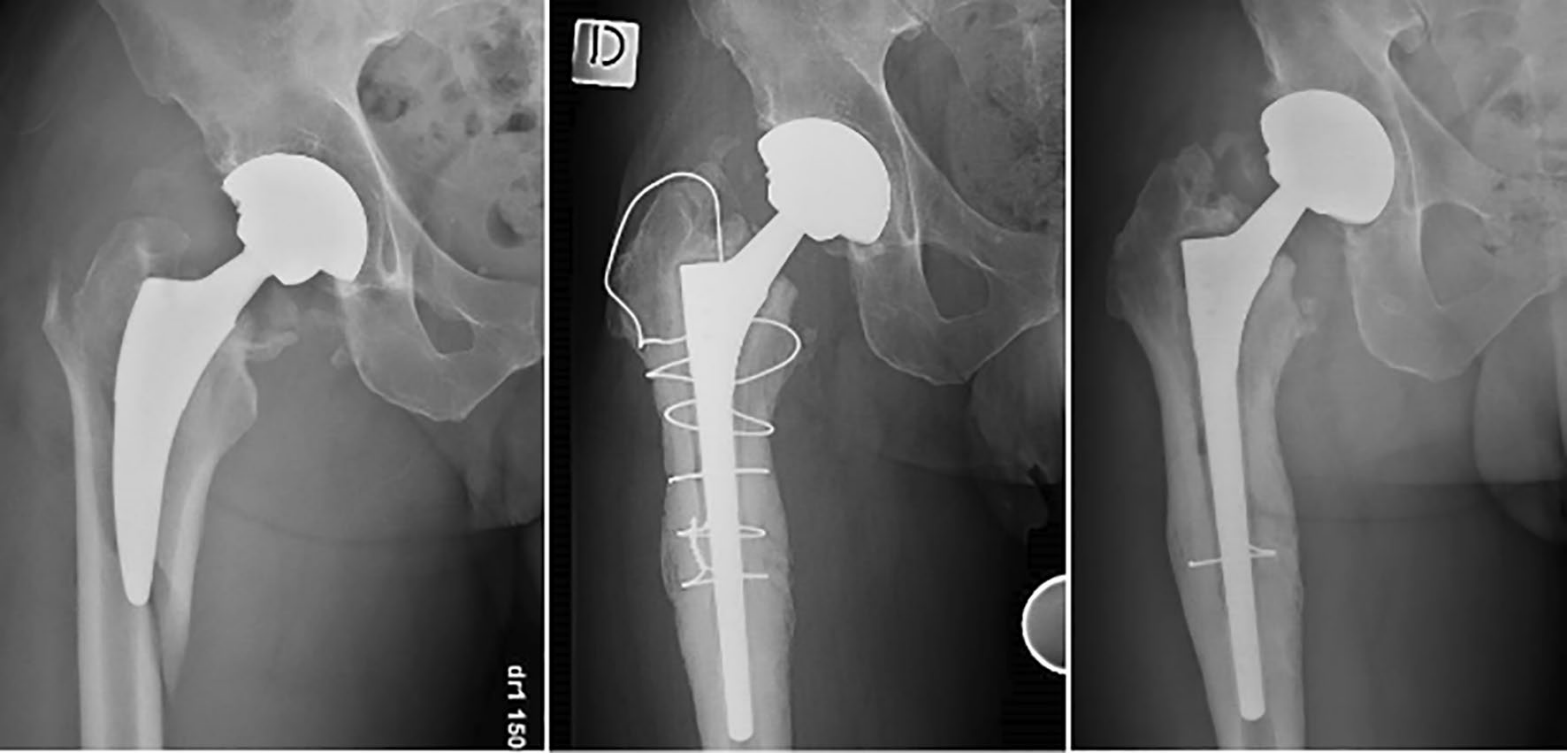
Radiographic analysis of a sample case with final cerclage removal performed after 21 months from indexed surgery

Six (5.3%) patients underwent cerclage removal for minimal tight pain (Fig. 2).

### Outcome evaluation

**Fig. 3:**
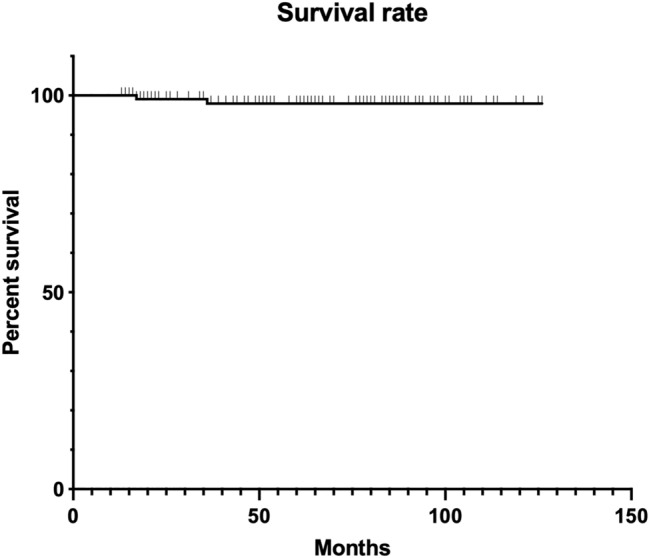
Kaplan–Meier survival function for the failure of Wagner stem (*n* = 114) in patients treated with stem revision for PPFx

The stem-related revision rate for any cause at the final FU was 1.7%, due to PJI (Fig. 3).

No patients underwent stem revision surgery for aseptic loosening. Fracture healed in all the included patients with a union-rate of 100%.

**Fig. 4:**
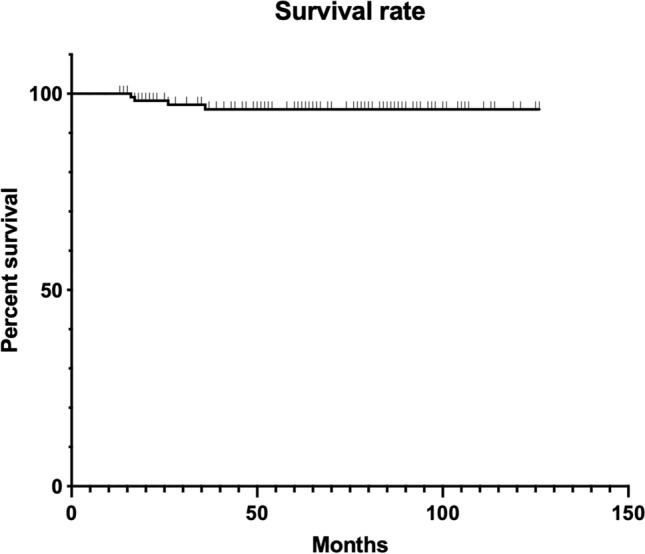
Kaplan–Meier survival function for the failure of fixed compontents (*n* = 114) in patients treated with stem revision for PPFx

The re-operation rate for any cause was 9.6%, with an implant survival rate for overall failure of 96.5% (Fig. 4).

## Discussion

PPFx are one of the most feared complications after THA. PPFx are the third most common reasons for revision after THA and the second most common reason for revision after the fourth year from the primary THA according to the Swedish National Hip Arthroplasty Register.

PPFx management includes several unique points. First, the surgical management requires expertise both in fracture fixation and in revision arthroplasty. Moreover, since these fractures usually occur in elderly population, a comprehensive evaluation of the patient is of paramount importance to choose the most tailored treatment. A complete and multidisciplinary evaluation including fracture pattern, previous situation of THA (failing prosthesis), type of stem, medical comorbidities and bone quality is mandatory [16].

Up to now, the Vancouver classification is the most widely used system to describe these fractures and to address surgical management. Several other algorithms have been proposed (Coventry classification, Unified Classification System) but they still need further validation [17–19].

The Vancouver classification is based on the following three pillars: fracture location, stem stability and bone stock preservation. Although some B1 fractures (short oblique or transverse at the tip of the stem) might benefit from stem revision [20], basically this procedure is required when the femoral implant is loose (Type B2 and B3 fractures). Stem stability should be accurately checked in the preoperative planification but is a common experience that the final decision on stem stability is mainly an intraoperative judgment. Some reports underline that up to 20% of loose stems remain undiagnosed at radiographic-based approach and up to 47% of B2 fractures, confirmed intraoperatively, were initially classified as B1 on X-ray evaluation [21, 22]. Recently, Aubert et al. defined possible risk factors associated with an early femorotomy during a revision stem procedure. The authors identified bracket sign (defined as a distal spot weld between the surface of the implant and closest endosteum), bone contact in zones 2, 6, 12, and 13 as predictors of a stable stem requiring an early femorotomy for stem removal [23]. Such parameters should be clearly identified in the preoperative analysis but final decision whether revise or not is still intraoperative. According to these findings, the authors always checked the intraoperative stability of the stem in all the included patients.

Generally, long stems alone or in addition with plate or allografts are required for B2 fracture management. A recent survey among members of the European Hip Society (EHS) showed strong consensus in treating Vancouver B2 fractures with stem revision e cerclages [24].Kahn et al. performed a systematic review of 22 studies on B2 fracture management. Out of 343 fractures, 298 were treated with stem revision and 45 with open reduction and internal fixation (ORIF) alone. Higher reoperation rate was observed in the non-revised cohort of patients [25]. The Swedish National Hip Arthroplasty Register support this evidence showing a reoperation rate of 32% in patients treated with ORIF alone compared to a 10% and 23% rate in patients managed with stem revision alone or stem revision and ORIF, respectively.

On the contrary, some papers showed good results with ORIF alone in Vancouver B 2 PPF [26–28]. Joestl et al. analyzed a cohort of 36 patients, with 8 of them treated with ORIF utilizing a locking compression plate. The authors demonstrated optimal results with 100% of union rate in the ORIF group without secondary stem migration. In the postoperative period, the ORIF group has limited weight bearing for the first 6 weeks while the revision group was allowed to partial of full weight bearing as tolerated [28]. A recent review confirmed that successful outcomes could be obtained without the need for revising a loose stem [29]. Although these recent data, is the authors opinion that stem removal and revision of a loose component is of a paramount importance. This procedure combined with a stable fracture reduction allows an early mobilization and rapid recovery of full weight bearing. Since the majority of the patients are old and affected by multiple comorbidities, early mobilization and rapid recovery of self-reliance and independence is fundamental to prevent further adverse events and avoiding stasis-related complications (i.e., pressure ulcers, pulmonary thromboembolism, deep vein thrombosis, pneumonia) [30, 31].

From the technical point of view, a long conical tapered stem was implanted in all patients. Wagner SL (Zimmer-Biomet, Warsaw, IN) is a diaphyseal fitting revision stem which is made of a titanium–aluminum–niobium alloy with a rough‑blasted surface [32]. The shaft of the prosthesis has a conus angle of 2° and eight longitudinal ribs around the stem. The stem is available in four lengths (190, 225, 265 and 305 cm) and one neck-shaft angle (135°). In the setting of B2 PPFx, this stem achieves optimal results bridging the fracture and providing a biomechanically sound in-axis load-sharing. The ribs of the stem provide rotational stability to the implant, while the taper provides axial stability.

The choice of the proper stem length and size during the preoperative planning guarantees an adequate load-sharing avoiding stress risers that could jeopardize fracture healing and stem stability.

After stem stability is obtained bypassing the fracture level by at least two cortical diameters, fracture is easily reduced and stabilized with simple metal cerclages adopting a distal-to-proximal sequence (from the diaphysis to the grater trochanter). From a biomechanical point of view, sound stem stability provides resistance to axial and rotational stability (stem ribs) while cerclage wires offer a good resistance to bending forces making bridging locking plates not necessary in most cases.

As Frangie et al. demonstrated in a cadaveric case–control study [33], there is no difference in stem and fracture stability in reducing first the fracture and then implanting the stem, compared to a “ream-first” technique (first stem implantation and then fragments reduction).

Even if anatomical reduction is always advisable and should be obtained whenever possible, the proposed technique allows to achieve a complete fracture healing with optimal clinical results even in case of minimal fracture displacement if axis, length, and rotation of the femoral diaphysis is obtained.

The presented results favorably compare with the available literature on this topic [22, 28]. Baum et al. [34] provided results from a cohort of 59 patients (35 treated with revision and 24 with ORIF). They reported 12.5% of fracture recurrence in the ORIF group and 28.6% implant-related complications in revision group. In a recent meta-analysis comparing ORIF and revision procedure in B2 PPFX, Lewis et al. [35] reported an overall complication rate of 24% for ORIF versus 18% for revision group. In the presented cohort, 6 patients sustained a new traumatic PPFx (3 greater trochanter fractures and 3 type C fractures). In all cases the previous fracture was healed, and the stem was well fixed. One patient underwent reoperation for fracture displacement at one month after the indexed surgery. Fixation with additional wires was performed.

Financial implications of these injuries have also been investigated. Philips et al. [36] found that the mean cost of treating 146 PPFx was £23.649 with the ward cost being responsible for 80.3% of the total cost. Obviously, the mean implant cost is higher for revision arthroplasty patients, but the quicker rehabilitation led to a reduction in general complication rate, length of stay and overall lower costs. Considering the growing rate of PPFx, the proposed technique should be considered to reduce the economic burden of such clinical scenario.

Undoubtedly, this study has several limitations. The retrospective nature of the analysis contains inherent limitations which must be considered when evaluating the results. The absence of control groups made any considerations on different treatment options not possible. The main limitation of the proposed technique is the extension of the fracture over the femoral isthmus. In infra-isthmus PPFx a sound stem stability is difficult to obtain. Such situation benefits from a stable bridge plating according to standard osteosynthesis techniques. However, the prospective collection of data, the relatively long follow-up, the fact that all the patients underwent a standardized protocol of surgical treatment and follow-up can be considered strengths of this study.

## Conclusions

PPFx are one of the most challenging fractures not only from the surgical point of view but also for the complexity of patient’s features. The management requires trauma and revision arthroplasty skills, and a multidisciplinary approach should be standard of care. More intraoperative surgical solutions and implants must always be available.

The presented standard and reproducible surgical technique obtain optimal clinical and radiological results with limited complication rate at mid-term follow up. Preoperative planning as well as careful intraoperative surgical technique are of a paramount importance. Further high-quality long-term studies are needed to directly compare different techniques, define limits and correct pitfalls.

## References

[1] Berry DJ (1999). Epidemiology: hip and knee. Orthop Clin N Am.

[2] Marsland D, Mears SC (2012). A Review of periprosthetic femoral fractures associated with total hip arthroplasty. Geriatr Orthop Surg Rehabil..

[3] Boylan MR, Riesgo AM, Paulino CB (2018). mortality following periprosthetic proximal femoral fractures versus native hip fractures. J Bone Jt Surg Am.

[4] Bhattacharyya T, Chang D, Meigs JB, Estok DM, Malchau H (2007). Mortality after periprosthetic fracture of the femur. J Bone Jt Surg Am.

[5] Cook RE, Jenkins PJ, Walmsley PJ, Patton JT, Robinson CM (2008). Risk factors for periprosthetic fractures of the hip: a survivorship analysis. Clin Orthop Relat Res.

[6] Lindahl H, Oden A, Garellick G, Malchau H (2007). The excess mortality due to periprosthetic femur fracture. A study from the Swedish national hip arthroplasty register. Bone.

[7] Schmidt AH, Kyle RF (2002). Periprosthetic fractures of the femur. Orthop Clin N Am.

[8] Duncan CP, Masri BA (1995). Fractures of the femur after hip replacement. Instr Course Lect.

[9] Masri BA, Meek RM, Duncan CP (2004). Periprosthetic fractures evaluation and treatment. Clin Orthop Relat Res.

[10] Giannoudis PV, Kanakaris NK, Tsiridis E (2007). Principles of internal fixation and selection of implants for periprosthetic femoral fractures. Injury.

[11] Abdel MP, Cottino U, Mabry TM (2015). Management of periprosthetic femoral fractures following total hip arthroplasty: a review. Int Orthop.

[12] Fink B (2014). Revision arthroplasty in periprosthetic fractures of the proximal femur. Oper Orthop Traumatol.

[13] Gruen TA, McNeice GM, Amstutz HC (1979). Modes of failure of cemented stem-type femoral components: a radiographic analysis of loosening. Clin Orthop Relat Res.

[14] Kevin TH, Timothy BA, Albert OG (2015). In brief: classifications in brief: Brooker classification of heterotopic ossification after total hip arthroplasty. Clin Orthop Relat Res.

[15] Cavagnaro L, Chiarlone F, Divano S, Capello AG, Felli L, Burastero G (2019). Primary cementless stems in septic hip revision: Indications and results. J Orthop Surg.

[16] Haddad FS (2020). Periprosthetic fractures: more challenges ahead. Bone Jt J..

[17] Ninan TM, Costa ML, Krikler SJ (2007). Classification of femoral periprosthetic fractures. Injury.

[18] Unified Classification System for Periprosthetic Fractures (UCPF) (2018) J Orthop Trauma 32 : S141-S144. 10.1097/BOT.0000000000001068. 10.1097/BOT.0000000000001068 29256962

[19] Duncan CP, Haddad FS (2014). The Unified Classification System (UCS): Imporving our understanding of periprosthetic fractures. Bone Jt J.

[20] Tsiridis E, Krikler S, Giannoudis, (2007). PV. Periprosthetic femoral fractures: current aspects of management. Injury.

[21] Fleischman AN, Chen AF (2015). Periprosthetic fractures around the femoral stem: overcoming challenges and avoiding pitfalls. Ann Transl Med.

[22] Lindahl H, Garellick G, Regnér H, Herberts P, Malchau H (2006). Three hundred and twenty-one periprosthetic femoral fractures. J Bone Jt Surg Am.

[23] Aubert T, Auberger G, Gerard P, Lhotellier L, Marmor S, Graff W (2022). Risk factors associated with femorotomy or fracture during cementless stem removal and generation of an individual predictive risk score. J Arthroplasty.

[24] Thaler M, Weiss C, Lechner R, Epinette JA, Karachalios TS, Zagra L (2023). (2023) Treatment of periprosthetic femoral fractures following total hip arthroplasty: results of an online survey of the European Hip Society. Hip Int.

[25] Khan T, Grindlay D, Ollivere BJ, Scammell BE, Manktelow AR, Pearson RG (2017). A systematic review of Vancouver B2 and B3 periprosthetic femoral fractures. Bone Jt J..

[26] Flury A, Hasler J, Pagenstert G (2021). Open reduction and internal fixation might be a valuable alternative to stem revision in Vancouver B2 periprosthetic femoral fractures, irrespective of the stem’s design. Arch Orthop Trauma Surg.

[27] Martinov S, D’ulisseHaumont SE (2022). Comparative study of Vancouver type B2 periprosthetic fractures treated by internal fixation versus stem revision. Arch Orthop Trauma Surg.

[28] Joestl J, Hofbauer M, Lang N, Tiefenboeck T, Hajdu S (2016). Locking compression plate versus revision-prosthesis for Vancouver type B2 periprosthetic femoral fractures after total hip arthroplasty. Injury.

[29] Stoffel K, Blauth M, Joeris A, Blumenthal A, Rometsch E (2020). Fracture fixation versus revision arthroplasty in Vancouver type B2 and B3 periprosthetic femoral fractures: a systematic review. Arch Orthop Trauma Surg.

[30] Parker M, Johansen A (2006). Hip fracture. BMJ.

[31] Craik RL (1994). Disability following hip fracture. Phys Ther.

[32] Wagner H (1993) Post graduate lectures of the first European Federation of National Associations of Orthopaedics and Traumatology (EFORT). Masson, Paris. Revision of femoral stem with important loss of bone stock, pp 64–74

[33] Frangie R, Han S, Noble PC, Gold JE, Lanfermeijer ND, Reddy KI, Ismaily SK, Su J, Schroder SJ, Rodriguez-Quintana D (2023). The stability of fixation of Vancouver B2 periprosthetic femoral fractures: effect of implantation technique. J Arthroplasty.

[34] Baum C, Leimbacher M, Kriechling P, Platz A, Cadosch D (2019). Treatment of periprosthetic femoral fractures Vancouver type B2: revision arthroplasty versus open reduction and internal fixation with locking compression plate. Geriatr Orthop Surg Rehabil..

[35] Lewis DP, Tarrant SM, Cornford L, Balogh ZJ (2022). Management of Vancouver B2 periprosthetic femoral fractures, revision total hip arthroplasty versus open reduction and internal fixation: a systematic review and meta-analysis. J Orthop Trauma.

[36] Phillips JR, Boulton C, Morac CG, Manktelov AR (2011). What is the financial cost of treating periprosthetic hip fractures?. Injury.

